# Whose Issue Is It Anyway? The Effects of Leader Gender and Equality Message Framing on Men’s and Women’s Mobilization Toward Workplace Gender Equality

**DOI:** 10.3389/fpsyg.2018.02497

**Published:** 2018-12-11

**Authors:** Stephanie L. Hardacre, Emina Subašić

**Affiliations:** School of Psychology, University of Newcastle, Callaghan, NSW, Australia

**Keywords:** gender equality, leadership, solidarity action, social change, social identity, collective action, legitimacy, message framing

## Abstract

Social psychologists have not fully investigated the role of leadership in mobilizing widespread support for social change, particularly gender equality. The burden of achieving gender equality is typically placed on women (particularly female leaders) – the main targets of such inequality. Traditional approaches frame workplace gender equality as either a *women’s issue*, which limits men’s (non-target’s) involvement in the movement, or a *meritocratic non-issue* that exists due to women’s (target’s) tendency to pursue less intensive careers. In contrast to such work focusing on women’s experiences as targets of discrimination or men’s role in preserving inequality, we propose a solidarity-based approach that positions men and women as *agents of change*. This approach relies on two processes: leadership processes – particularly leadership as a form of influence based on shared identities among leaders and followers (e.g., their gender group); and political solidarity as a way to mobilize the silent majority (men) to work as allies beside a minority (women) and embrace equality as a common cause for *both* groups. In two experiments (*Ns* = 338, 336) we studied how leader gender and message framing affect men’s and women’s support for equality by contrasting a solidarity-based framing of gender equality as a common cause for men and women, with a women’s issue frame (Experiment 1) or a meritocratic frame (Experiment 2). The statement was attributed to a male or female leader (Experiments 1–2) or, additionally, to a government agency (Experiment 1). Women reported higher sense of common cause (Experiment 2) and collective action intentions than men (Experiments 1–2), and higher intentions under common cause compared to meritocracy frames (Experiment 2). Interestingly, male leaders invoked higher sense of common cause and collective action intentions for both men *and* women regardless of framing (Experiment 2). Irrespective of leader gender however, as predicted common cause framing boosted perceived leader prototypicality, legitimacy, and influence across the board (Experiments 1–2). Yet this was qualified by women (compared to men) rating leaders as more legitimate and influential under common cause compared to meritocracy framing (Experiment 2). Women’s reactions to equality messages, and the intersection of leadership and solidarity toward equality are discussed.

## Introduction

The burden of achieving gender equality has traditionally been placed on women (particularly female leaders), who are usually the main targets of such inequality (Rindfleish and Sheridan, 2003). Typical approaches and responses to gender inequality tend to frame the issue as either the responsibility of women alone to address (e.g., ‘women’s work’; Mavin, 2008), or as a meritocratic ‘non-issue’ existing only due to women’s tendency to embark on less demanding education and career trajectories (Whelan, 2013). Placing the responsibility on women alone (as both women’s issue and meritocratic frames do) alleviates men’s prerogative to support women affected by inequality and provides them ample rationalization to abstain from doing so (Becker and Barreto, 2014). Meritocratic frames of gender equality imply that so long as individuals work hard, they should measure up favorably against necessary employment criteria and subsequently succeed in the workplace (Williams, 2015). When used as an explanation for why gender inequality exists, they have been shown to reduce men’s understanding of inequality (de Vries, 2010) and decrease the likelihood of women acting collectively against it (Major et al., 2002).

In contrast to work focusing on women’s experiences as targets of discrimination or men’s role in maintaining inequality, in this paper we take a political solidarity-based approach using common cause message framing. Such framing utilizes inclusive language that emphasizes solidarity between men and women and makes salient (leaders’ and) followers’ shared social identity (Fiol et al., 1999). This solidarity-based approach positions both men and women as ‘agents of change’ in a concerted effort to engage a broader audience of women *and* men (i.e., targets and non-targets; see Subašić et al., 2018). The political solidarity model (Subašić et al., 2008) conceptualizes social change as a process through which members of a majority (e.g., men) challenge the authority (e.g., male-dominated systems) in solidarity with the minority (e.g., women). In contrast to traditional frames of men as perpetrators and women as victims, this approach positions gender equality as a common cause for men and women to address together – as “comrades in struggle” (hooks, 1984, p. 67). This approach relies on two key processes. Firstly, leadership and influence processes based on shared social identity with those seeking to advance social change. The second process involves the concept of political solidarity as a way of mobilizing the silent majority (i.e., men as an over-represented group within the workplace) to work as allies alongside a minority (i.e., women as an under-represented group) and embrace gender equality as a common cause for *both* groups (i.e., men and women; Subašić et al., 2018).^1^

In line with these ideas, Seyranian (2014) found that within a renewable energy context, leaders who highlighted shared grievances of the collective group were evaluated as more prototypical, effective, trustworthy, and persuasive, and inspired greater collective action among their male and female followers. Wiley et al. (2012) also found that men were more likely to participate in collective action if they believed that many men supported gender equality, which common cause framing infers. Finally, Subašić et al. (2018) demonstrated that framing gender equality as a common cause for both men and women (rather than a women’s issue) heightened men’s *and* women’s collective action intentions. However, while women were mobilized by both male and female leaders, men were mobilized primarily by male leaders who espoused a common cause message (and less so by male leaders who focused on gender equality as a women’s issue). This research demonstrates that not only does it matter *what* is being said (i.e., the message frame), but also *who* is saying it (i.e., the leader) and to *whom* (i.e., the target; see also Subašić et al., 2012). To the extent leaders can foster a sense of common cause or solidarity among followers by realigning their personal self-interests with broader collective goals, collective mobilization can be expected (Turner et al., 2008).

This sense of common cause refers to men’s and women’s feelings of solidarity with those women affected by gender inequality. It involves sharing similar viewpoints, values, concerns, and goals with those people who object to and seek to reduce gender-based inequality (Subašić et al., 2018). This sense of common cause (and shared identity) most readily arises when leaders and followers share a salient in-group (e.g., their gender group; Haslam et al., 2011; Wiley et al., 2012). Indeed, by enhancing self-categorical bonds between themselves and their relevant in-group, in-group leaders are more effective than outgroup leaders at influencing followers (Duck and Fielding, 2003). Finally, because gender is one of the most salient in-groups (Fiske, 1998), and arguably at its *most* salient within gender inequality contexts, people are not only conscious of their own gender in such contexts but also whether those leading the charge toward equality are men or women. Yet research has largely neglected the intricacies of gender and leadership when examining when and why female (and male) equality leaders might mobilize support for gender equality (Powell, 1990).

Increased awareness of leader gender can negatively affect female equality leaders because they suffer particular disadvantage within masculine organizational contexts due to prejudicial evaluations regarding their competency (Eagly and Carli, 2003). Moreover, when female leaders *do* adopt masculine behaviors (i.e., those seen as prototypical of leaders), they violate communal expectations of women and face backlash effects (Okimoto and Brescoll, 2010). Women also face accusations of self-interest (de Vries, 2015). This can destabilize their social change efforts (Eagly et al., 1978), with female leaders typically being perceived as less legitimate and influential compared to their male counterparts who face no such accusations (Drury and Kaiser, 2014). Feminists in general also face widespread stigmatization which can delegitimize their calls for equality (Kamen, 1991). For example, Anastosopoulos and Desmarais (2015) found undergraduates evaluated a job candidate less positively when she identified as a feminist, and feminist women are typically viewed as angry, unattractive, man-hating extremists (Faludi, 1991).

In contrast, male leaders and feminist men receive more favorable evaluations (Eagly and Carli, 2003; Anderson, 2009) and encounter positive reactions when drawing attention to gender inequality (Rasinski and Czopp, 2010). However, while feminist men are viewed more positively than feminist women, they are also perceived as less stereotypically masculine or heterosexual, which can affect their readiness to identify as feminists and participate in equality efforts (Anderson, 2009). Yet sexism confrontations by men are more successful than those by women because men are seen as acting counter to group interests and as having something to lose, ultimately affording them greater legitimacy than female leaders (Czopp and Monteith, 2003; Drury and Kaiser, 2014). Certainly, Cihangir et al. (2014) found that suggestions of sexism by male sources were more beneficial to female targets than suggestions by female sources (e.g., targets exhibited increased self-confidence and greater likelihood of filing a complaint). Alternatively, Drury (2013) discovered that female observers of sexism confrontations were unaffected by confronter gender, which makes sense given confrontations by either gender aim to elevate women’s social status.

Therefore it seems an asymmetry exists regarding male versus female leaders’ capacity to mobilize men’s and women’s support for gender equality (Subašić et al., 2018). To examine this idea, we extend Subašić et al. (2018) work in a novel way by assessing the psychological processes underlying leader influence and measuring whether participants’ attitudes and evaluations of those leading the charge for equality differ based on leaders’ gender. However, just as focusing exclusively on women is inadequate for achieving equality, viewing male leaders’ engagement as the panacea for inequality is equally naïve (de Vries, 2015). Accordingly, the present research examines the role of leader gender and solidarity-based message framing in mobilizing support for gender equality by men and women, to determine under what conditions these factors do or *do not* affect mobilization toward equality.

In two experiments, we use manipulation statements attributed to either a male or female leader (Experiments 1–2) to examine whether the gender of the leader affects their capacity to mobilize support for equality, as extant literature suggests (e.g., Seyranian, 2014; Subašić et al., 2018). In Experiment 1, we additionally attribute the statement to a gender-neutral control (i.e., a government agency), against which the effects and impact of leader *gender* can be compared. It was hoped that inclusion of this control would serve as a valid baseline, allowing us to further investigate participants’ responses to male and female leaders relative to a non-gendered control condition (further extending Subašić et al., 2018). We also contrast solidarity-based frames of gender equality as a common cause with traditional approaches framing equality as a women’s only issue (Experiment 1) or a meritocratic issue (Experiment 2), to determine whether the way in which the equality message is framed affects support for equality. We focus on two sets of outcome variables: mobilization variables [including collective action intentions (Experiments 1–2) and sense of common cause (Experiment 2)], and leadership variables [including leader prototypicality, legitimacy, and influence (Experiments 1–2)].

In line with Seyranian (2014), we hypothesize that when gender equality is framed as a common cause rather than a women’s issue (Experiment 1) or a meritocratic issue (Experiment 2), men and women will report higher collective action intentions and sense of common cause (Hypothesis 1a). Similarly, we also predict that when gender equality is framed as a common cause rather than a women’s issue (Experiment 1) or a meritocratic issue (Experiment 2), men and women will evaluate leaders as being more prototypical, legitimate, and influential (Hypothesis 1b). Finally, as per Subašić et al. (2018), we hypothesize that while women’s collective action intentions and sense of common cause will remain stable regardless of who promotes equality, men’s intentions and sense of common cause will be higher when the equality message is attributed to a male leader rather than a female leader (Experiments 1–2) or a government agency (Experiment 1), especially under common cause compared to women’s issue (Experiment 1) or meritocratic messages (Experiment 2; Hypothesis 2).

## Experiment 1

### Methods

#### Participants and Design

Participants were students at a large Australian university or members of the general public (*N* = 480, 240 women), between 17 and 68 years (*M_age_* = 26.37, *SD* = 9.41). They were recruited online via Facebook or Reddit (72%), or the university’s research participation program (28%). The results did not differ between these groups. Participants comprised 44% Australians, 35.8% Americans, 5.4% Canadians, 5.2% English, and 9.6% other. They were employed on a full- (33.5%), part-time (18.5%), or casual (17.9%) basis, or identified as unemployed (26.76%) or other (3.3%). Sixty-one percent were studying full- (50.2%) or part-time (8.8%) domestically, or full-time internationally (1.7%), with the remaining 39% not currently studying. The study was a 2 (participant gender: male, female) × 3 (leader gender: male leader, female leader, government agency) × 2 (message framing: women’s issue, common cause) factorial design, with equal numbers of men and women being allocated at random to one of six conditions.

An effect size of approximately *r* = 0.15 is typical in the field of psychology, which is equivalent to a partial eta-squared ($\eta_{p}^{2}$) of 0.0225 (Cafri et al., 2010). Thus an *a priori* statistical power analysis using Faul et al. (2007) G^∗^Power 3 program revealed that for a power of 0.80 (α = 0.05) the minimum sample to detect a small effect size of $\eta_{p}^{2}$= 0.0225 (or *f* = 0.151) using a 2 × 3 × 2 ANOVA is 422 (35 participants per cell). We increased this to 480 (40 per cell) to reach sufficient power after the anticipated exclusion of participants who failed the leader gender manipulation check. Sensitivity power analyses revealed that our actual obtained sample size (338) had the power to detect effect sizes of: $\eta_{p}^{2}$= 0.0228 (or *f* = 0.152) for the participant gender and message framing main effects and participant gender × message framing interaction, and $\eta_{p}^{2}$= 0.0280 (or *f* = 0.169) for the leader gender main effect and all remaining two- and three-way interactions.

#### Procedure and Materials

Participants completed a 15-min online questionnaire containing the experimental manipulations and dependent measures described below (full materials can be found in the Supplementary Material). The study was conducted in accordance with the principles and recommendations of the National Statement on Ethical Conduct in Human Research (2007), as per the University of Newcastle’s Human Research Ethics Committee. The protocol was approved by the University of Newcastle’s Human Research Ethics Committee (Protocol Number: H-2015-0143), which is affiliated with the National Health and Medical Research Council of Australia. All participants gave electronic informed consent in accordance with the Declaration of Helsinki. Participants were debriefed and offered the opportunity to withdraw.

##### Leader gender and message framing manipulations

A one-page press release ostensibly detailed the Gender Equality Commission[er]’s formation of a new group whose goal was to “address gender-based discrimination, sexual harassment, and other barriers to gender equality.” The vignette described gender inequality (e.g., “Women continue to earn less than men for equal work, and are less likely to be promoted to leadership positions compared to men”), and the group’s progress toward their goal in an annual report (e.g., “increase the number of women in leadership positions within companies and decrease the gender pay gap”). Leader gender (male, female, government agency) was manipulated by changing the Commission[er]’s name (e.g., “Margaret [Matthew] Jamieson” vs. “The Commission”) and using relevant pronouns (e.g., “her [his, our], she [he, it]”). Message framing (women’s issue, common cause) was manipulated via equality group name (e.g., “[Men and] Women for Gender Equality”) and message content (e.g., “it is vital [men and] women are engaged and committed to tackling this issue [together],” “[men and boys] working [together] with women and girls”). The Commission[er] communicated their pledge “to serve the [men and] women of this world” and stated their group “builds on the excellent work of all those [men and] women currently committed to achieving gender equality.”

##### Manipulation checks

All measures used 7-point Likert scales (1 = strongly disagree/not at all, 4 = neither agree nor disagree/somewhat, 7 = strongly agree/very much so). To assess the manipulation’s success, participants identified the Commission[er]’s gender (male/female/not stated), and rated the extent to which the vignette provided information regarding inequality being (a) a women’s only issue or (b) a common cause for men and women.

##### Collective action intentions

Eight items (α = 0.95) measured participants’ collective action intentions supporting gender equality (adapted from van Zomeren et al., 2004; Glasford and Calcagno, 2012). Example items included: “[Imagine you were approached by the Commission and asked to participate in their latest campaign for gender equality. In response, would you be willing to…] Sign a petition to stop inequality against women,” “Talk to male [female] colleagues about gender inequality.”

##### Leader prototypicality

Five items (α = 0.85) measured participants’ perceived prototypicality of the leader (adapted from Platow and van Knippenberg, 2001). For example, “[Thinking of the gender equality movement and people who support it, would you say the Commission:] Is representative of members of the movement,” and “Stands for what people in the movement have in common.”

##### Leader legitimacy

Four purpose-built items assessed the leader’s perceived legitimacy (“…do you think the Gender Equality Commission’s statement was Legitimate/Justified/Valid/Reasonable”; α = 0.96).

##### Leader influence

Four items measured the leader’s perceived influence (adapted from Wiley et al., 2012; “…do you think the Gender Equality Commission’s statement was Persuasive/Convincing/Compelling/Credible”; α = 0.92).

### Results

SPSS Version 23 was used to perform between-participants ANOVA’s on all dependent variables, with participant gender, leader gender, and message framing as factors.

#### Manipulation Checks

Frequency statistics confirmed that 70% of participants correctly identified the Commission[er]’s gender (68.1% male, 72.5% female, 70% not stated). Participants who failed to correctly identify the leader’s gender were excluded from further analyses, bringing the final sample to 338 (167 women). Participant exclusion distribution rates did not differ significantly by condition [χ(5) = 6.321, p = 0.276], and are reported alongside final participant gender distributions for each cell in Table 1.

**Table 1:** Participant exclusion distribution rates and final participant gender distribution numbers by condition, based on participants who failed the leader gender manipulation check.

	% of participants	Number of male	Number of female	Number of overall
	who failed the	participants	participants	participants
Condition	manipulation check	remaining	remaining	remaining
Male leader, women’s issue	25	30	30	60
Male leader, common cause	38.75	28	21	49
Female leader, women’s issue	25	26	34	60
Female leader, common cause	30	27	29	56
Government agency, women’s issue	33.75	28	25	53
Government agency, common cause	25	32	28	60
Totals	30	171	167	338

*The third and fourth columns represent the number of male and female participants remaining in each condition following the exclusion of those participants who failed the leader gender manipulation check.*

Participants in the women’s issue conditions were significantly more likely than participants in the common cause conditions to agree that the article discussed “The need for women alone to stand up for equality” and “Inequality being a women’s only issue” [F(1,336) = 55.986, p < 0.001, $\eta_{p}^{2}$= 0.143; Ms = 3.80 and 2.53, SDs = 1.60 and 1.50, respectively). In contrast, participants in the common cause conditions were significantly more likely than participants in the women’s issue conditions to agree that the article discussed “The need for both men and women to stand up for equality” and “Inequality being a men’s and women’s issue” [F(1,336) = 109.870, p < 0.001, $\eta_{p}^{2}$ = 0.246; Ms = 5.90 and 4.06, SDs = 1.40 and 1.80, respectively). No other significant effects were found, indicating that our manipulations were successful.

#### Correlations

Inspection of the correlations assessing relationships between the dependent variables indicated that these were measured reliably and are consistent with existing research (see Table 2).

**Table 2:** Means, standard deviations, and correlations (Spearman’s rho) among study variables.

Dependent variable	*M*	*SD*	Leader prototypicality	Leader legitimacy	Leader influence
Collective action intentions	4.63	1.79	0.261^∗∗^	0.548^∗∗^	0.526^∗∗^
Leader prototypicality	4.56	1.05		0.559^∗∗^	0.597^∗∗^
Leader legitimacy	4.95	1.73			0.783^∗∗^
Leader influence	4.18	1.53			

*N = 338. ^∗∗^*p* = 0.01 (two-tailed).*

#### Mobilization Variables

As reported below, contrary to Hypothesis 1a neither men nor women reported higher collective action intentions under common cause (compared to women’s issue) framing. Additionally, Hypothesis 2, which predicted that men (but not women) would report higher intentions under male leaders (compared to female or government leaders), particularly under common cause messages, was not supported. Instead, men (and women) reported similar collective action intentions irrespective of leader gender and message framing.

##### Collective action intentions

Absence of a significant main effect of message framing failed to provide support for Hypothesis 1a, which predicted that men and women would report higher intentions under common cause compared to women’s issue framing. Instead, participants reported similar collective action intentions regardless of how the message was framed [*M*_commoncause_ = 4.73, *SD* = 1.68; *M*_women’sissue_ = 4.52, *SD* = 1.88; *F*(1,326) = 2.10, *p* = 0.148, $\eta_{p}^{2}$= 0.006].

Our three-way prediction that men would report higher collective action intentions under male leaders, particularly under common cause messages (H2), was not supported, *F*(2,326) = 0.753, *p* = 0.472, $\eta_{p}^{2}$= 0.005.

Finally, a significant main effect of gender revealed that women (*M* = 5.23, *SD* = 1.61) expressed higher collective action intentions than men (*M* = 4.03, *SD* = 1.75), *F*(1,326) = 45.176, *p* < 0.001, $\eta_{p}^{2}$= 0.122. All other main effects and interactions were non-significant, all *F* ≤ 0.718, *ps* ≥ 0.489, $\eta_{p}^{2}$≤ 0.004.

##### Leadership variables

Supporting Hypothesis 1b, all participants consistently rated leaders as being significantly more prototypical, legitimate, and influential when leaders framed gender equality as a common cause for men and women to work toward together, as opposed to an issue concerning women alone (reported below).

##### Leader prototypicality

A main effect of message framing revealed that in line with Hypothesis 1b, participants perceived leaders as being significantly more prototypical of the gender equality movement when they promoted common cause (*M* = 4.71, *SD* = 0.98) rather than women’s issue framing (*M* = 4.43, *SD* = 1.11), *F*(1,326) = 5.972, *p =* 0.015, $\eta_{p}^{2}$= 0.018. None of the remaining main effects or interactions reached significance, all *F* ≤ 2.373, *p*s ≥ 0.095, $\eta_{p}^{2}$≤ 0.014.

##### Leader legitimacy

Supporting Hypothesis 1b, a main effect of message framing demonstrated that participants viewed leaders as being significantly more legitimate when they promoted common cause (*M* = 5.17, *SD* = 1.55) rather than women’s issue framing (*M* = 4.75, *SD* = 1.87), *F*(1,326) = 5.874, *p* = 0.016, $\eta_{p}^{2}$= 0.018. A main effect of gender also showed that women (*M* = 5.26, *SD* = 1.62) perceived leaders to be significantly more legitimate than men did (*M* = 4.66, *SD* = 1.79), *F*(1,326) = 10.304, *p* < 0.001, $\eta_{p}^{2}$= 0.031. All other main effects and interactions were non-significant, all *F* ≤ 1.151, *p*s ≥ 0.318, $\eta_{p}^{2}$≤ 0.007.

##### Leader influence

Replicating all other leadership evaluation findings and supporting Hypothesis 1b, participants perceived leaders to be significantly more influential when they promoted gender equality as a common cause (*M* = 4.40, *SD* = 1.44) compared to a women’s issue (*M* = 3.98, *SD* = 1.58), *F*(1,326) = 7.355, *p* = 0.007, $\eta_{p}^{2}$= 0.022. Similar to our leader legitimacy results, a main effect of gender again showed that women (*M* = 4.52, *SD* = 1.39) rated leaders as more influential than men did (*M* = 3.84, *SD* = 1.58), *F*(1,326) = 18.028, *p* < 0.001, $\eta_{p}^{2}$= 0.052. No other main effects or interactions were significant, all *F* ≤ 0.932, *p*s ≥ 0.395, $\eta_{p}^{2}$≤ 0.006.

### Discussion

Experiment 1 saw gender equality being promoted by either a male or a female leader, or a gender-neutral government agency, and framed as either a common cause for men and women to combat, or as an issue concerning women alone. Overall, women reported higher collective action intentions than men (addressed in the section “General Discussion”). However, our prediction that framing equality as a common cause (rather than a women’s issue) would result in increased mobilization toward equality (H1a) was not supported. Instead, men and women reported equal collective action intentions irrespective of how equality was promoted. This is in contrast to Subašić et al. (2018), who found common cause framing heightened participants’ collective action intent (although for men, this effect only emerged when a male leader promoted the common cause message). Indeed, a key aim was to examine whether the source of the gender equality message being a male leader (compared to a female or government leader) would increase men’s mobilization toward equality, particularly under common cause messages (H2). However, this hypothesis was not supported. Instead, men and women expressed similar collective action intentions irrespective of who promoted the equality message and how.

While our collective action findings do not reflect Subašić et al. (2018), the present work extends theirs in a novel way by explicitly examining the leadership and influence processes underlying participants’ mobilization. Importantly, our prediction that solidarity-based common cause frames of gender equality would elicit more positive evaluations of leaders (as per Seyranian, 2014; H1b) was supported. Indeed, when leaders highlighted equality as a common cause rather than a women’s issue, participants consistently perceived those leaders as being significantly more prototypical, legitimate, and influential – a pattern which emerged irrespective of leader gender. These novel findings are addressed in the section “General Discussion.”

## Experiment 2

Experiment 2 aimed to build upon Experiment 1 (and Subašić et al.’s 2018 paper) and manipulate the perceived legitimacy of inequality by contrasting common cause framing with meritocratic framing. In contrast to traditional women’s issue approaches which subtly place the responsibility for addressing inequality onto women, meritocracy framing more blatantly assigns the blame for inequality to women. Indeed, meritocratic ideology preserves workplace inequality by implying it is partly women’s fault due to their tendency to pursue less intensive career and education paths (Whelan, 2013). Such ideology argues that so long as women gain the necessary experience, they should climb the meritocratic hierarchy with ease (Cech and Blair-Loy, 2010). This framing echoes Sandberg’s (2013) ‘lean in’ philosophy, which maintains that if only women would show up and “sit at the table” (p. 27), learn to master negotiation techniques, take advantage of mentorship and leadership opportunities, and commit to their own individual growth, they would succeed in the workplace. Essentially, this kind of meritocratic framing legitimizes gender inequality by foisting blame onto the individual failings of people, rather than considering discriminatory structural factors that genuinely undermine the achievement of equality (Major and Schmader, 2001). Understandably then, meritocracy is often proffered as an argument or excuse for abolishing affirmative action policies such as quotas or preferential treatment strategies which take into account minority or under-represented group status, because these strategies are perceived as violating meritocratic principles (Son Hing et al., 2002).

Meritocratic justifications of gender inequality are thus particularly troublesome given that the perceived illegitimacy of gender inequality is a key predictor for participation in collective action (van Zomeren et al., 2008). Indeed, the more one perceives gender inequality to be unjust or illegitimate, the higher one’s likelihood of participating in collective action, and vice versa (van Zomeren et al., 2008). Certainly, unquestioning adherence to meritocratic ideals is known to undermine men’s understanding of gender inequality (de Vries, 2010), and decrease women’s likelihood of acting collectively against inequality (Major et al., 2002). For example, Jetten et al. (2011) found that higher perceived legitimacy and pervasiveness appraisals of discrimination were linked to lowered collective action intentions among women in academia. McCoy and Major (2007) also showed that priming meritocratic beliefs among women (e.g., “effort leads to prosperity,” p. 343) resulted in them justifying group disadvantage by reducing their perceptions of discrimination. Similarly, men and women were more likely to accept gender inequality following exposure to essentialist theories of social change, such as the belief that gender-based labor segregation is due to innate biological differences between men and women (Morton et al., 2009). However, these studies relied on either providing false feedback regarding fellow female employee’s legitimacy appraisals, or simply priming meritocratic and essentialist beliefs, rather than explicitly manipulating the suggested reasons behind gender inequality’s existence.

In contrast, study designs that *do* experimentally manipulate the perceived legitimacy of gender inequality and measure the effects on individuals’ mobilization allow for the assumed causal direction to be tested (van Zomeren et al., 2008). Accordingly, Experiment 2 saw workplace inequality being framed either as a common cause for men and women to work toward together, or as an issue existing due to meritocratic reasons. By explicitly manipulating the perceived legitimacy of gender inequality, we hoped to examine the effects that legitimacy appraisals or explanations have on men’s and (particularly) women’s responses to calls for gender equality. Additionally, we expected that contrasting common cause framing with a more polarizing version of women’s issue framing (i.e., meritocracy) would strengthen the effects of common cause framing on participants’ mobilization. Indeed, implying that inequality exists for legitimate reasons further absolves men of any responsibility to combat it (Whelan, 2013).

Furthermore, inclusion of the government agency in Experiment 1 may have contributed to the flattening of responses we observed on our leader gender factor. Due to this, and given the importance of leadership processes to mobilization and our desire to determine the effects of leader *gender* on mobilization, we focused solely on male and female leaders in Experiment 2. A lack of statistical power in Experiment 1 might further explain our lack of significant findings, given 30% of participants were excluded due to failing the leader gender manipulation check. This resulted in Experiment 1’s cell size decreasing from the recruited 40 participants per cell to an average of only 28 participants per cell. Consequently, we improved Experiment 2’s power by increasing the sample size from 40 to 45 per cell. We also measured participants’ sense of common cause (i.e., solidarity; Subašić et al., 2018), given solidarity is of key importance to the present paper. This measure seeks to better assess men’s and women’s sense of solidarity with those women affected by gender inequality. Finally, belief in meritocracy is a core American ideology (Kluegel and Smith, 1986), therefore an American sample was used as it was presumed meritocratic explanations of inequality would be most familiar to Americans, regardless of whether they themselves endorse the ideology (McCoy and Major, 2007).

### Methods

#### Participants and Design

Participants were 360 White Americans (180 women), aged 18–65 years (*M_age_* = 34.13, *SD* = 11.66), who were recruited via crowdsourcing website Prolific (2017) and remunerated $1.15 USD. Prolific allows recruitment of naïve participants based on specified criteria (e.g., employment status), and use of such crowdsourcing portals efficiently and appropriately produces data with similarly good reliability as found in typical undergraduate samples (Behrend et al., 2011). Participants were employed on a full- (63.9%), part-time (18.3%), self-employed (13.6%), casual (2.2%), or other (1.9%) basis. Students comprised 19.4% of the sample, while 80.6% were not currently studying. The study followed a 2 (participant gender: male, female) × 2 (leader gender: male leader, female leader) × 2 (message framing: meritocratic issue, common cause) factorial design with equal numbers of men and women being randomly allocated to one of the four conditions.

A G^∗^Power analysis revealed that for a power of 0.80 (α = 0.05), the minimum sample to detect a small effect size of $\eta_{p}^{2}$= 0.0225 (or *f* = 0.151) using a 2 × 2 × 2 ANOVA should be 343 participants (approximately 42 per cell). We increased this to 360 (45 per cell) to obtain sufficient power following the expected removal of those who failed the leader gender manipulation check. Sensitivity power analyses showed that our obtained sample size (336 participants) had the power to detect effect sizes of $\eta_{p}^{2}$= 0.0228 (or *f* = 0.152) for all main effects and interactions.

#### Procedure and Materials

Participants completed a 15-min online questionnaire following the same procedure as in Experiment 1. The full materials can be found in the Supplementary Material.

##### Leader gender and message framing manipulations

We imbued Experiment 2’s vignette with an increased emphasis on corporate culture depictions of workplace inequality issues, given our sample consisted primarily of employed participants who presumably had greater workplace experience compared to Experiment 1’s sample, which consisted primarily of younger students (M_age_ = 26.37, SD = 9.41; 61% studying; 52% employed). Accordingly, although leader gender (male, female) was manipulated in the same manner as in Experiment 1 (“Margaret [Matthew],” “her [his]”), the Gender Equality Commissioner was replaced with the Chief Delegate to the Organization for Economic Co-Operation and Development. Additionally, in both message framing conditions, the Chief Delegate first described their aspirations to address pay and leadership disparities within the business and corporate world in particular (e.g., “increase the number of women in business leadership positions,” “women still comprise only 21% of board members and 9% of CEOs globally”).

Our message framing manipulation consisted of one additional paragraph that framed inequality as either an issue that primarily exists due to meritocratic reasons and that women can overcome so long as they exert sufficient effort in the workplace (meritocratic issue), or a common cause for both men and women to address together (common cause). The meritocratic manipulation paragraph stated: “While gender inequality continues to be a significant social and economic issue, those women who are in senior management roles show that it is possible to move up the leadership ladder by working hard, ‘leaning in,’ and making sacrifices. These women demonstrate that all individuals can succeed in the workplace irrespective of their gender — as long as they are prepared to invest the time, energy, and significant effort needed for such advancement. Indeed, in the business world, those who apply themselves and make sacrifices along the way reap the rewards, because business — and society more broadly — has always rewarded hard work.” The common cause manipulation stated “While gender inequality continues to be a significant social and economic issue, it is now an issue that matters to both men and women. However, our report shows that progress toward this common goal has stalled, which is why it’s important that both parties are engaged and committed to tackling this issue together. Admittedly, while there is no ‘silver bullet,’ we know that men and boys working together with women and girls to promote gender equality contributes to achieving a host of health and developmental outcomes, not just those within the business world.”

##### Manipulation checks

Participants identified the gender of the Chief Delegate (male/female), then rated the extent to which inequality was discussed as (a) a meritocratic issue or (b) a common cause.

##### Collective action intentions

Six items assessed participants’ collective action intentions toward achieving gender equality (α = 0.91; adapted from Calogero, 2013; Subašić et al., 2018). Sample items included: “[Imagine that the Chief Delegate has approached you directly to help with their campaign for gender equality. In that context, please rate the extent to which you agree with the following statements…] Sign a petition (in person or online) in support of women’s rights and gender equality,” “I would vote for a political party that fights against gender inequality.”

##### Sense of common cause

Four items measured participants’ sense of common cause (i.e., solidarity) with those women affected by gender inequality (α = 0.96; adapted from Subašić et al., 2018). Sample items included: “Those seeking to reduce income inequality and leadership disparities between men and women share my goals and concerns,” “I feel solidarity with the women affected by income inequality and leadership disparities,” and “I see myself as someone who shares the views of the women who object to these forms of inequality.”

##### Leadership measures

Measures of leader prototypicality (α = 0.95), legitimacy (α = 0.95), and influence (α = 0.95) were identical to those used in Experiment 1.

### Results

To investigate the effects of message framing on men’s and women’s responses, significant participant gender × message framing interactions were unpacked by performing separate one-way ANOVA’s on relevant dependent variables at both levels of participant gender.

#### Manipulation Checks

Frequency statistics revealed 93% of participants identified the Chief Delegate’s gender correctly (95.6% male, 91.1% female). The 24 participants (7%) who failed this check were excluded from further analyses, hence the final sample comprised 336 (170 women). Participant exclusion distribution rates did not differ significantly by Condition [χ(3) = 3.571, p = 0.312] and are reported below in Table 3 alongside final participant gender distributions for each cell. The higher percentage of participants passing the leader gender check relative to Experiment 1 is likely due to participants being remunerated via Prolific, which allows recruitment of participants who have a track record of serious study attempts (e.g., successful study completion rates over 85%).

**Table 3:** Participant exclusion distribution rates and final participant gender distribution numbers by condition, based on participants who failed the leader gender manipulation check.

	% of participants	Number of male	Number of female	Number of overall
	who failed the	participants	participants	participants
Condition	manipulation check	remaining	remaining	remaining
Male leader, merit issue	5.55	42	43	85
Male leader, common cause	3.33	43	44	87
Female leader, merit issue	10	41	40	81
Female leader, common cause	7.77	40	43	83
Totals	7	166	170	336

*The third and fourth columns represent the number of male and female participants remaining in each condition following the exclusion of those participants who failed the leader gender manipulation check.*

Participants in the meritocracy conditions were significantly more likely than those in the common cause conditions to agree that the article discussed “Women in senior management roles showing it’s possible to move up the leadership ladder by working hard” and “The idea that all individuals can succeed in the workplace irrespective of their gender, as long as they work hard” [F(1,328) = 176.954, p < 0.001, $\eta_{p}^{2}$= 0.350; Ms = 5.83 and 3.53, SDs = 1.60 and 1.27, respectively). Participants in the common cause conditions were significantly more likely than those in the meritocracy conditions to agree that the article discussed “The need for men and women to be engaged and committed to tackling gender inequality together” and “The need for men and boys to work together with women and girls to promote gender equality” [F(1,328) = 317.891, p < 0.001, $\eta_{p}^{2}$= 0.492; Ms = 6.14 and 3.21, SDs = 1.17 and 1.82]. There was also a participant gender × message framing interaction [F(1,328) = 9.693, p = 0.002, $\eta_{p}^{2}$= 0.029], with simple effects performed at each level of message framing showing only a main effect of gender for merit conditions, F(1,164) = 8.495, p = 0.004, $\eta_{p}^{2}$= 0.049. Women were significantly less likely to agree with the common cause manipulation items (M = 2.81, SD = 1.72) than men (M = 3.61, SD = 1.85), indicating that women were more capable of distinguishing between the message frames. No other significant effects were observed, indicating our message framing manipulation was successful.

#### Correlations

Table 4 shows that the correlations between the dependent variables were again consistent with extant research.

**Table 4:** Means, standard deviations, and correlations (Spearman’s rho) among study variables.

Dependent variable	*M*	*SD*	Sense of common cause	Leader prototypicality	Leader legitimacy	Leader influence
Collective action intentions	4.68	1.62	0.787^∗∗^	0.037	0.220^∗∗^	0.153^∗^
Sense of common cause	5.17	1.56		0.043	0.238^∗∗^	0.156^∗∗^
Leader prototypicality	4.86	1.41			0.608^∗∗^	0.671^∗∗^
Leader legitimacy	5.20	1.48				0.808^∗∗^
Leader influence	4.69	1.45				

*N = 336. ^∗^*p* = 0.05, ^∗∗^*p* = 0.01 (two-tailed).*

#### Mobilization Variables

Hypothesis 1a predicted that men and women would report higher collective action intentions and sense of common cause under common cause compared to meritocracy message frames. Providing partial support for this hypothesis, women (but not men) reported higher intentions (but not sense of common cause) under common cause framing. Additionally, Hypothesis 2 was not supported, which predicted that men would report higher intentions and sense of common cause under male leaders who promoted a common cause message. Instead, men reported significantly higher collective action intentions and sense of common cause under male (compared to female) leaders irrespective of message framing. Importantly, women also reported higher intentions and sense of common cause under the same conditions.

##### Collective action intentions

**FIGURE 1:**
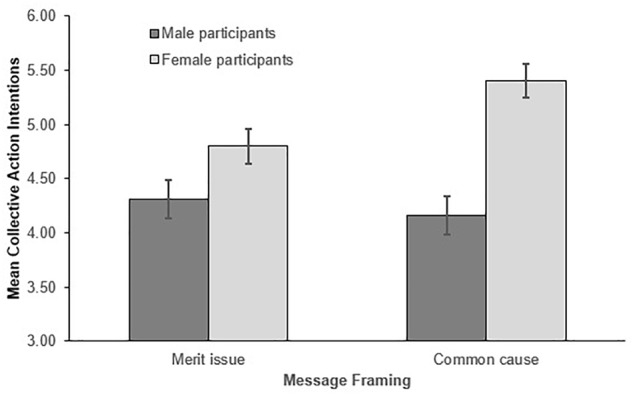
Mean collective action intentions as a function of message framing and participant gender. Error bars represent the standard errors.

Contrary to Hypothesis 1a, no significant main effect of message framing was found, with participants instead expressing similar collective action intentions irrespective of how the message was framed [M_commoncause_ = 4.78, SD = 1.72; M_merit issue_ = 4.55, SD = 1.50; F(1,328) = 1.78, p = 0.185, $\eta_{p}^{2}$= 0.005]. However, we detected a significant participant gender × message framing interaction [shown in Figure 1; F(1,328) = 5.035, p = 0.026, $\eta_{p}^{2}$= 0.015], which qualified the significant main effect of gender that was also detected (M_women_ = 5.13, SD = 1.46; M_male_ = 4.28, SD = 1.61), F(1,328) = 26.404, p < 0.001, $\eta_{p}^{2}$= 0.075.

Simple effects performed at both levels of participant gender revealed a significant main effect of message framing for women, *F*(1,168) = 7.322, *p* = 0.008, $\eta_{p}^{2}$= 0.042, but not men, *F*(1,164) = 0.342, *p* = 0.560, $\eta_{p}^{2}$= 0.002. Providing partial support for Hypothesis 1a (which predicted that men *and* women would report higher intentions under common cause frames), only women reported higher intentions under common cause (*M* = 5.40, *SD* = 1.44) compared to meritocracy frames (*M* = 4.80, *SD* = 1.46). Alternatively, contrary to Hypothesis 1a, men expressed similar (albeit still lower than women’s) collective action intentions regardless of how the equality message was framed (*M_merit issue_* = 4.31, *SD* = 1.50; *M_commoncause_* = 4.16, *SD* = 1.76).

Finally, absence of a significant three-way interaction failed to provide support for Hypothesis 2 which predicted that men would report higher intentions under male leaders who promoted a common cause message, *F*(1,328) = 0.480, *p* = 0.489, $\eta_{p}^{2}$= 0.001. Instead, a significant leader gender main effect showed that irrespective of how the equality message was framed, male (and female) participants expressed significantly higher collective action intentions when male leaders discussed equality (*M* = 4.86, *SD* = 1.60) compared to when female leaders did (*M* = 4.49, *SD* = 1.62), *F*(1,328) = 4.816, *p* = 0.029, $\eta_{p}^{2}$= 0.014. This indicates that male (compared to female) leaders were better at mobilizing male *and* female participants. All remaining main effects and interactions were non-significant, all *F* ≤ 1.766, *p*s ≥ 0.185, $\eta_{p}^{2}$≤ 0.005.

##### Sense of common cause

No significant main effect of message framing was found, thus failing to support Hypothesis 1a. Instead, participants reported similar sense of common cause regardless of how the message was framed [(*M_commoncause_* = 5.25, *SD* = 1.68; *M_merit issue_* = 5.09, *SD* = 1.43; *F*(1,328) = 0.65, *p* = 0.419, $\eta_{p}^{2}$= 0.002].

Absence of a significant three-way interaction again failed to support Hypothesis 2 which predicted that men would report higher sense of common cause under male leaders promoting a common cause message, *F*(1,328) = 0.899, *p* = 0.344, $\eta_{p}^{2}$= 0.003. Instead, replicating our collective action findings, a significant main effect of leader gender revealed that irrespective of message framing, men *and* women reported significantly higher sense of common cause under male leaders (*M* = 5.33, *SD* = 1.46) than female leaders [*M* = 5.00, *SD* = 1.65; *F*(1,328) = 4.429, *p* = 0.036, $\eta_{p}^{2}$= 0.013]. We also observed a significant main effect of gender, with women (*M* = 5.78, *SD* = 1.17) expressing higher sense of common cause than men (*M* = 4.55, *SD* = 1.67), *F*(1,328) = 63.457, *p* < 0.001, $\eta_{p}^{2}$= 0.162. No other significant main effects or interactions were detected, all *F* ≤ 3.279, *p*s ≥ 0.071, $\eta_{p}^{2}$≤ 0.010.

#### Leadership Variables

Supporting Hypothesis 1b and replicating Experiment 1’s significant findings, participants evaluated leaders as being significantly higher in leader prototypicality, legitimacy, and influence when they promoted gender equality as a common cause rather than a meritocratic issue. However, this was qualified by an interaction showing that women in particular rated leaders as significantly more legitimate and influential under common cause compared to meritocracy framing.

##### Leader prototypicality

Consistent with Hypothesis 1b, leaders who promoted equality as a common cause (M = 5.42, SD = 0.99) were evaluated as being significantly more prototypical of the gender equality movement than leaders who used meritocratic explanations for inequality (M = 4.29, SD = 1.54), F(1,328) = 65.527, p < 0.001, $\eta_{p}^{2}$= 0.167. A significant leader gender main effect also revealed that female leaders (M = 5.12, SD = 1.34) were rated as being significantly more prototypical of the gender equality movement than male leaders (M = 4.62, SD = 1.43), F(1,328) = 12.437, p < 0.001, $\eta_{p}^{2}$= 0.037. No other main effects or interactions were detected, all F ≤ 2.051, ps ≥ 0.153, $\eta_{p}^{2}$≤ 0.006.

##### Leader legitimacy

**FIGURE 2:**
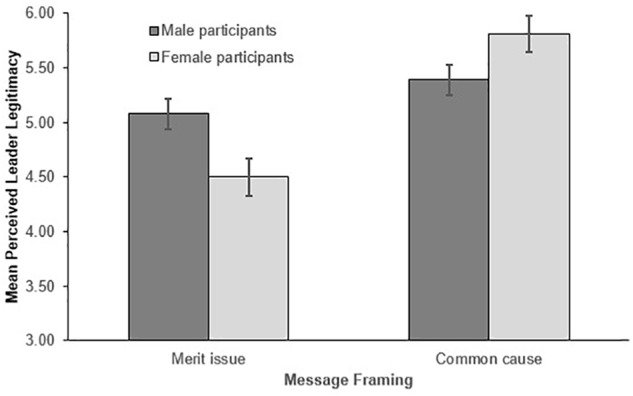
Mean perceived leader legitimacy as a function of message framing and participant gender. Error bars represent the standard errors.

Consistent with Hypothesis 1b, a significant main effect of message framing showed that leaders who employed common cause framing (M = 5.61, SD = 1.20) were viewed as significantly more legitimate than leaders who relied on meritocracy framing (M = 4.79, SD = 1.63), F(1,328) = 28.006, p < 0.001, $\eta_{p}^{2}$= 0.079. However, this finding was qualified by the significant two-way interaction between participant gender and message framing shown in Figure 2, F(1,328) = 10.553, p = 0.001, $\eta_{p}^{2}$= 0.031. Simple effects performed at each level of participant gender showed a significant main effect of message framing for women, F(1,168) = 31.613, p < 0.001, $\eta_{p}^{2}$= 0.158, but not men, F(1,164) = 2.576, p = 0.110, $\eta_{p}^{2}$= 0.015. Women evaluated leaders as significantly less legitimate when they framed equality as a meritocratic issue (M = 4.50, SD = 1.82), rather than a common cause for men and women (M = 5.81, SD = 1.18). In contrast, men viewed leaders as being equally legitimate regardless of how they framed their equality message (M_commoncause_ = 5.39, SD = 1.19; M_merit issue_ = 5.08, SD = 1.36). No other main effects or interactions were significant, all F ≤ 1.389, ps ≥ 0.239, $\eta_{p}^{2}$≤ 0.004.

##### Leader influence

**FIGURE 3:**
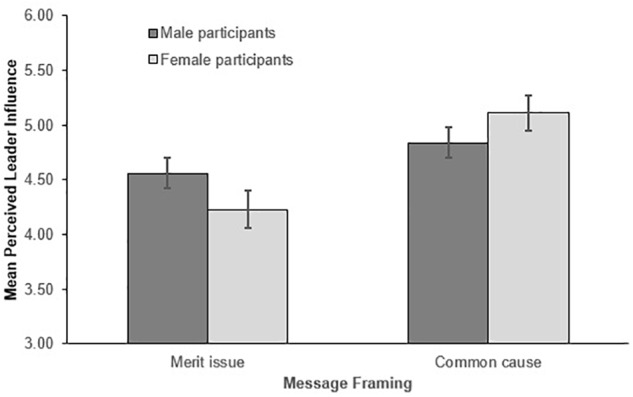
Mean perceived leader influence as a function of message framing and participant gender. Error bars represent the standard errors.

Supporting Hypothesis 1b, and replicating our prototypicality and legitimacy findings, leaders who promoted gender equality as a common cause (*M* = 4.98, *SD* = 1.29) were considered significantly more influential than those who promoted it as an issue pertaining to meritocracy (*M* = 4.39, *SD* = 1.55), *F*(1,328) = 14.347, *p* < 0.001, $\eta_{p}^{2}$= 0.042. However, in line with our legitimacy results, this finding was again qualified by a significant participant gender × message framing interaction, *F*(1,328) = 3.857, *p* = 0.050, $\eta_{p}^{2}$= 0.012 (see Figure 3). Simple effects examining both levels of participant gender showed message framing had a significant effect on women, *F*(1,168) = 13.932, *p* < 0.001, $\eta_{p}^{2}$= 0.077, but not men, *F*(1,164) = 2.028, *p* = 0.156, $\eta_{p}^{2}$= 0.012. Replicating our leader legitimacy findings, women viewed leaders as significantly more influential when they framed equality as a common cause (*M* = 5.11, *SD* = 1.39) rather than an issue of merit (*M* = 4.23, *SD* = 1.69). Again reflecting our leader legitimacy findings, men perceived leaders as being equally influential regardless of how they promoted equality (*M_commoncause_* = 4.84, *SD* = 1.17; *M_merit issue_* = 4.56, *SD* = 1.38).

### Discussion

A key aim of Experiment 2 was to directly contrast male and female equality leaders (bar a gender-neutral control) to better determine whether they differ in their capacity to mobilize individuals toward gender equality. Supporting Hypothesis 1b and replicating Experiment 1’s findings, participants again evaluated leaders as being significantly more prototypical, legitimate, and influential under common cause rather than meritocratic framing. However, this finding was qualified by an interaction which showed that women (but not men) evaluated all leaders as being significantly more legitimate and influential when they promoted common cause instead of meritocracy frames. These findings are addressed in the section “General Discussion.”

Another key aim of Experiment 2 was to examine how manipulating the perceived legitimacy of gender inequality affects men’s and women’s responses to the issue, by contrasting common cause framing with meritocratic framing. Replicating Experiment 1’s significant findings, women reported significantly higher collective action intentions than men, and the same pattern was found for women’s sense of common cause with the women affected by inequality. While Hypothesis 1a was not supported in Experiment 1, in the current experiment women reported significantly higher collective action intentions (but not higher sense of common cause) under common cause compared to meritocracy message frames. Meanwhile, men’s mobilization remained unaffected by message framing. Therefore, despite all participants evaluating leaders who promoted common cause frames more positively, Hypothesis 1a was partially supported for women, but not men, and only for collective action intentions, not sense of common cause. Additionally, in regards to Hypothesis 2, men (*and* women) expressed higher collective action intentions and higher sense of common cause under male leaders compared to female leaders. This indicates that male leaders were more successful than female leaders at mobilizing male *and* female participants. However contrary to Hypothesis 2 this effect was *not* enhanced under common cause messages. These findings are discussed below.

As anticipated, contrasting solidarity-based common cause framing with a more polarizing and legitimating version of traditional women’s issue frames (i.e., meritocracy) strengthened the effects of such framing on (women’s) mobilization. One limitation is that including a third women’s issue condition would have allowed us to better determine the effects of common cause framing relative to meritocratic framing. Nevertheless, these results indicate that women, as the primary targets of gender inequality (and as compared to men, who are typically non-targets and even perpetrators of inequality) are *particularly* sensitive to how the issue of equality is promoted, and remain differentially affected by legitimating meritocratic messages. Certainly, women’s adoption of meritocratic beliefs surrounding inequality can lead them to “reconstruct the glass ceilings they have cracked” (Cech and Blair-Loy, 2010, p. 371). Our findings reflect this, given that women were significantly *less* likely to report collective action intentions or feelings of common cause under meritocratic frames. Essentially, providing women with a meritocratic explanation of inequality removed their motivation to agitate for collective action, likely as a reaction to the message’s legitimating content. Ultimately, discrimination perceived as legitimate removes the impetus for collective action by “undermining the validity of the collective grievances of the group” (Jetten et al., 2011, p. 118).

## General Discussion

This paper extends Subašić et al. (2018) findings by explicitly examining the role of leadership and influence processes in affecting social change. As predicted (H1b), across both studies common cause framing (compared to more traditional frames of equality) enhanced leadership evaluations of all leaders irrespective of their gender. Indeed, common cause leaders were evaluated as being significantly more prototypical, legitimate, and influential by both men and women (Experiments 1–2). This indicates that solidarity-based common cause framing plays a key role in affecting support for social change toward equality. As Steffens et al. (2014) assert, “leaders need not only to ‘be one of us’…but also to ‘do it for us’…to ‘craft a sense of us’…and to ‘embed a sense of us”’ (p. 1001). Common cause framing achieves this perception of leaders being ‘one of us’ by making them appear more prototypical and subsequently more legitimate and influential to followers. Certainly, prototypical leaders derive their influence partly from perceptions that they embody collective group interests, which common cause framing achieves (van Knippenberg, 2011). When (male and female) leaders position themselves as a common leader for men *and* women and thus craft a sense of common cause and shared identity, both men and women appear more favorable toward, and receptive of, these equality leaders.

Despite this, our prediction that common cause framing would also result in higher collective action intentions and sense of common cause on behalf of both men and women was not wholly supported (H1a). Instead, providing partial support for Hypothesis 1a, women (but not men) expressed increased collective action intentions (but not sense of common cause) under common cause messages compared to meritocratic messages (Experiment 2). Meanwhile, men appear less affected by *what* is being said, compared to *who* is saying it: message framing did not affect men’s mobilization in either experiment, but in Experiment 2 they (along with women) reported higher collective action intentions and sense of common cause under male leaders – irrespective of how they framed the issue. However, because this effect was not enhanced under common cause messages, our prediction that men would report higher intentions and sense of common cause under male leaders promoting common cause messages was not supported (H2).

Nevertheless, this finding that male leaders mobilized participants more effectively than female leaders (Experiment 2) signals that leader gender remains a crucial aspect of the leader-influence process when striving to mobilize follower support toward social change. It is not sufficient to merely “walk the talk” (Kotter, 2007, p. 101) by promoting equality as a common cause for men and women – it appears leaders must also embody a shared identity with their followers. Indeed, the gender of the leader seems to greatly affect their capacity to rally supporters, with male leaders invoking significantly greater mobilization than female leaders irrespective of *how* they framed their message, or how positively *or* negatively they were evaluated as leaders (Experiment 2). Due to male feminists being free from the stigma associated with being a female feminist, this may have contributed to their higher mobilization of participants (Anderson, 2009). Additionally, Wiley et al. (2012) discovered that men exposed to positive (rather than negative) feminist portrayals demonstrated increased feminist solidarity and collective action intentions. A male leader publicly endorsing equality could be viewed by men as a positive feminist role model, likely allowing men to readily adopt feminist behaviors (i.e., collective action intentions). Certainly, it has become increasingly socially acceptable for male leaders and celebrities to publicly self-identify as feminists (e.g., Barack Obama, Justin Trudeau, and Ryan Gosling), but this acceptance is yet to extend to women (Crowe, 2018). Furthermore, seeing fellow gender in-group members promote equality likely diminished men’s status protection motives, in contrast to outgroup female members who likely threatened their status and thus decreased their willingness to combat the status quo (Branscombe, 1998).

Taken together, our mobilization results speak to there being different mobilization pathways for men and women, just as there exists “differing starting places and processes for women and men” (de Vries, 2010, p. 36) in their journey toward supporting gender equality. Namely, as the principal targets of workplace gender inequality, women appear particularly sensitive to the way in which leaders frame their equality messages, especially when such messages can be perceived as legitimating and therefore preserving gender inequality (e.g., meritocratic frames). Women are both demobilized by, and prone to negatively evaluating leaders who choose to adopt such legitimating messages. Furthermore, in both experiments women expressed significantly higher collective action intentions (and sense of common cause in Experiment 2) than men. This strong gender difference demonstrates that women, as the primary targets of gender inequality, are more readily invested in and mobilized for equality than are men. Certainly, women are highly motivated to act collectively against inequality because it damages their group’s prospects (Van Zomeren and Spears, 2009), and such feminist behavior aims to elevate women’s status relative to men, hence is likely more attractive to women than to men (Radke et al., 2018). This reflects extant work in related domains, for example workplace gender discrimination (Iyer and Ryan, 2009), sexism confrontations (Becker and Barreto, 2014), and women’s sexual objectification (Guizzo et al., 2017).

These results have implications for the study of social change toward gender equality, specifically in regards to leadership and shared identity. Namely, our findings suggest that men are doubly advantaged in mobilizing followers because they already possess a shared identity with both male and female followers: shared gender identity and dominant in-group membership with men, and shared cause (in the form of gender equality) with women (irrespective of how they frame the issue; Subašić et al., 2018). Essentially, male leaders signal to men *and* women that “we are all in this together” (Subašić et al., 2018, p. 7). In contrast, female leaders, who are admittedly fellow targets of inequality alongside their female followers or employees, do not yet possess a similar shared identity with their (male) followers. Future research should explore alternative message framing or leadership style strategies that female leaders could adopt in order to erode the clear disadvantage they face in gender equality contexts (and beyond).

### Limitations and Future Research

These results should be considered in light of certain limitations. Firstly, we did not replicate Subašić et al.’s (2018) finding that solidarity framing increased men’s and women’s collective action intent (an effect that only emerged for men when a male leader promoted the common cause message). One methodological explanation for this is potential weakness of our manipulation vignettes or the manipulation checks themselves. While in the correct rank order, responses of participants in the women’s issue conditions to the women’s issue manipulation checks in Experiment 1 were actually below the scale’s midpoint, indicating a ‘Neither agree nor disagree’ response. The Likert-type manipulation check items may not have adequately distinguished between message framing conditions, and additionally common cause condition participants might have misinterpreted and agreed with the women’s issue manipulation items too. Certainly, this condition ultimately encompassed equality as a women’s (*and* a men’s) issue. However, these lowered scores could also indicate disagreement that the article discussed inequality as being a women’s only issue, and thus weakness of our vignette. Certainly, our manipulation differed slightly from Subašić et al.’s (2018). Whereas their manipulation specified an *Australian-based* Gender Equality Commission, our vignette focused on a supposedly *global* context and authority figure, with absence of a relevant superordinate identity to provide a localized context or initial shared identity for participants to relate to (e.g., an Australian or American Commission). Given the central role that social identity has been shown to play in the current and extant work (e.g., van Zomeren et al., 2008; Banfield and Dovidio, 2013; Klandermans, 2014), future research should investigate whether the inclusion (or exclusion) of a more specific superordinate identity would differentially affect (a) participants’ ability to recall the manipulation’s contents, and (b) participants’ mobilization toward equality. For example, future studies could explicitly and orthogonally manipulate the salience of global versus American superordinate identities.

Admittedly, many of our dependent variable means also hung around the scale’s midpoint. Certainly, offering a middle response category can increase the likelihood of participants disproportionately adopting a midpoint response style (Weijters, 2006). Nevertheless, this raises concerns as to whether participants properly engaged with the study materials, and whether our manipulations elicited the desired effect. The large percentage of participants (30%) who failed to correctly identify the leader’s gender in Experiment 1 indicates our manipulations were perhaps too subtle for participants to effectively distinguish between the three leader gender conditions. Indeed, participants had minimal (fictitious) information to base their appraisals on (e.g., first names and pronouns only). Future work requires improvement of the vignettes’ clarity and strength to ensure the desired effect is elicited (e.g., additional biographical information, photographs, real-world leaders), and use of alternative manipulation checks, such as writing a short paragraph about the vignette’s contents immediately following its presentation (Evans et al., 2015). Future research should also reconsider use of midpoint labeling and utilize larger samples.

Additionally, Subašić et al. (2018) sample comprised primarily young Australian undergraduates, whereas we utilized a combined Australian and American undergraduate and general public sample (Experiment 1) and an older American employed sample (Experiment 2). Thus participants’ personal experience (or lack thereof) of gender inequality may have differed, subsequently affecting their responses to different gender equality messages. Indeed, Experiment 2 used a largely employed American sample, and compared to typical undergraduate samples these working respondents had more likely been exposed to workplace gender inequality. Such familiarity could undermine women’s acceptance of the meritocratic ideology used, given employed women are more likely than men or unemployed women to be cognisant of structural inequalities and thus predisposed to interpret gender inequality as being structurally based (Cech and Blair-Loy, 2010). Despite attempts to keep the meritocracy messaging subtle, anecdotal feedback indicated some female participants did not ‘buy’ the meritocratic framing, particularly when attributed to female leaders (e.g., “I thought there was a subtle implication in Margaret’s statement that the barrier to women holding high level management positions was they weren’t working hard enough”; “It sounded like she was saying - other women can do it, so if you failed it’s your own fault and there is no systemic discrimination”). Future research could utilize more naïve samples and more nuanced meritocracy messages.

Our study design also limits the causal inferences we can draw. It is possible that the interventions used have the potential to be effective, however, were not intensive or long-lasting enough to engender concrete change in participants’ social change behaviors toward gender equality. The use of self-report measures also makes it difficult to ascertain whether collective action intentions translate into actual engagement with equality and feminism beyond the studies. Longitudinal studies directly engaging participants in collective action for equality could determine whether the effects of our manipulations extend beyond participation in the current studies. Furthermore, this could uncover whether participating in collective action can both *shape* individuals’ responses to inequality and *be shaped* by individuals’ perceptions and actions concerning inequality (Iyer and Ryan, 2009).

## Conclusion

Paradoxically, by virtue of their gender and the privileges it permits, male leaders seem to possess the ability to undertake gender equality leadership roles and mobilize men and women more effectively than female leaders (Marshall, 2007). Indeed, despite holding formal authority within the workplace, female leaders’ gender appears to limit their ability to address inequality (Martin and Meyerson, 1998). Yet we have also demonstrated that leaders’ influence and ability to mobilize follower support goes beyond their gender to encompass the rhetoric they adopt when discussing gender equality, in addition to *who* they are targeting. While women (compared to men) are inexorably more invested in, and thus more readily mobilized toward gender equality, they still remain particularly sensitive to how calls for equality are framed. This is in comparison to men, who appear relatively unaffected by differing frames of gender equality. Ultimately, the current studies point to the importance of there being an intersection between leadership and solidarity processes in order to bridge the gap between women’s and men’s mobilization toward gender equality. This intersection requires further unpacking to achieve a more nuanced understanding. Importantly, just as the present research highlights the existence of different mobilization pathways for targets and non-targets of workplace gender inequality, so too might there exist different pathways for male and female equality initiative leaders to achieve successful mobilization of followers.

## Author Contributions

SH assisted with the design, recruitment, analysis, and interpretation of Experiments 1 and 2, and additionally drafted the manuscript. ES was the primary supervisor of SH, assisted with the design, analysis, and interpretation of Experiments 1 and 2, and additionally provided theoretical and practical feedback on several drafts of the manuscript.

## Conflict of Interest Statement

The authors declare that the research was conducted in the absence of any commercial or financial relationships that could be construed as a potential conflict of interest.

## Footnotes

1 In this context the terms ‘minority’ and ‘majority’ are not referring exclusively to numerical categories but instead signify the social power available to women and men, in addition to their overall representativeness within the workplace and leadership positions. Thus women can be thought of as an under-represented group and men as an over-represented group (Subašić et al., 2008).
