# Supplementary material for: Whose Issue Is It Anyway? The Effects of Leader Gender and Equality Message Framing on Men’s and Women’s Mobilization Toward Workplace Gender Equality
Source: Front Psychol. 2018 Dec 11;9:2497. doi: 10.3389/fpsyg.2018.02497 (PMC6298257; doi:10.3389/fpsyg.2018.02497)
Supplement: Supplementary file 1 [file Table_1.DOC]

**Study 1 Manipulation Vignettes**

Leader Gender manipulations are underlined *Message Framing manipulations are italicised*

**Manipulation Vignette 1: Government Agency Framing Gender Inequality as a Women’s Issue**

**Please read the below information carefully as we will be asking you a series of questions about it.**

**Social Inequality Study**

As you may be aware, gender inequality remains a key issue worldwide, particularly within the workplace. Women continue to earn less than men for equal work, and are less likely to be promoted to leadership positions compared to men. Below is some information about the Gender Equality Commission and a recent campaign it has held to combat gender inequality.

**About the Gender Equality Commission**

The Gender Equality Commission was appointed in March 2015. The Commission’s role is to address gender-based discrimination, sexual harassment, and other barriers to gender equality *facing women* across the world. As part of its role, in 2015 the Commission created the *Women for Gender Equality* initiative which includes *50 of the world’s top female business and public sector leaders* striving to *achieve gender equality for women*. A milestone report has been released, detailing how the *Women for Gender Equality group* are faring with their aspirations to increase the number of women in leadership positions within companies and decrease the gender pay gap.

**The following is an excerpt from the Gender Equality Commission’s recent press release:**

**Gender Equality Commission Calls for Immediate Action: “The time to act is now”**

“As you know, the *Women for Gender Equality group* has just released its first annual report, *Gender Equality: What Matters to Women*. The research for the report involved *extensive consultation with women* across the world. Our key finding is that *gender equality matters to women* – but that progress towards *this goal* has stalled. Our report shows gender inequality continues to be a *significant social and economic issue facing women* across the world, which is why it is *vital women are engaged and committed to tackling this issue*.

The *Women for Gender Equality group* will continue to advocate for key reforms that reflect the priorities for *all women* at this time. It builds on the excellent work of *all those women currently committed* to achieving gender equality. While there is no ‘silver bullet’, we know that *working with women and girls* to promote gender equality contributes to achieving a host of health and developmental outcomes, not just those within the workplace.

We bring to our role as the Gender Equality Commission a sense of responsibility, obligation, and a deep commitment in our hearts to *serve the women of this world*, and strive to serve the gender equality movement to the best of our abilities. The time to act is now.”

Gender Equality Commission

March 2016

**Manipulation Vignette 2: Female Leader Framing Gender Inequality as a Women’s Issue**

**Please read the below information carefully as we will be asking you a series of questions about it.**

**Social Inequality Study**

As you may be aware, gender inequality remains a key issue worldwide, particularly within the workplace. Women continue to earn less than men for equal work, and are less likely to be promoted to leadership positions compared to men. Below is some information about the Gender Equality Commissioner and a recent campaign she has held to combat gender inequality.

**About the Gender Equality Commissioner**

Margaret Jamieson was appointed Gender Equality Commissioner in March 2015. The Commissioner’s role is to address gender-based discrimination, sexual harassment, and other barriers to gender equality *facing women* across the world. As part of her role, in 2015 the Commissioner created the *Women for Gender Equality* initiative which includes *50 of the world’s top female business and public sector leaders striving to achieve gender equality for women*. A milestone report has been released, detailing how the *Women for Gender Equality group* are faring with their aspirations to increase the number of women in leadership positions within companies and decrease the gender pay gap.

**The following is an excerpt from the Gender Equality Commissioner’s recent press release:**

**Gender Equality Commissioner Calls for Immediate Action: “The time to act is now”**

“As you know, the *Women for Gender Equality group* has just released its first annual report, *Gender Equality: What Matters to Women*. The research for the report involved *extensive consultation with women* across the world. Our key finding is that *gender equality matters to women* – but that progress towards *this goal* has stalled. Our report shows gender inequality continues to be a *significant social and economic issue facing women* across the world, which is why it is *vital women are engaged and committed to tackling this issue*.

The *Women for Gender Equality group* will continue to advocate for key reforms that reflect the priorities for *all women* at this time. It builds on the excellent work of *all those women currently committed* to achieving gender equality. While there is no ‘silver bullet’, we know that *working with women and girls* to promote gender equality contributes to achieving a host of health and developmental outcomes, not just those within the workplace.

I bring to my role as the Gender Equality Commissioner a sense of responsibility, obligation, and a deep commitment in my heart to *serve the women of this world*, and strive to serve the gender equality movement to the best of my abilities. The time to act is now.”


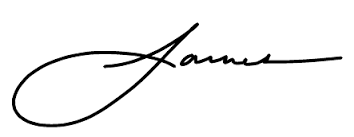


**Margaret Jamieson**

Gender Equality Commissioner

March 2016

**Manipulation Vignette 3: Male Leader Framing Gender Inequality as a Women’s Issue**

**Please read the below information carefully as we will be asking you a series of questions about it.**

**Social Inequality Study**

As you may be aware, gender inequality remains a key issue worldwide, particularly within the workplace. Women continue to earn less than men for equal work, and are less likely to be promoted to leadership positions compared to men. Below is some information about the Gender Equality Commissioner and a recent campaign he has held to combat gender inequality.

**About the Gender Equality Commissioner**

Matthew Jamieson was appointed Gender Equality Commissioner in March 2015. The Commissioner’s role is to address gender-based discrimination, sexual harassment, and other barriers to gender equality *facing women* across the world. As part of his role, in 2015 the Commissioner created the *Women for Gender Equality* initiative which includes *50 of the world’s top female business and public sector leaders striving to achieve gender equality for women*. A milestone report has been released, detailing how the *Women for Gender Equality group* are faring with their aspirations to increase the number of women in leadership positions within companies and decrease the gender pay gap.

**The following is an excerpt from the Gender Equality Commissioner’s recent press release:**

**Gender Equality Commissioner Calls for Immediate Action: “The time to act is now”**

“As you know, the *Women for Gender Equality group* has just released its first annual report, *Gender Equality: What Matters to Women.* The research for the report involved *extensive consultation with women* across the world. Our key finding is that *gender equality matters to women* – but that progress towards this goal has stalled. Our report shows gender inequality continues to be a *significant social and economic issue facing women* across the world, which is why it is *vital women are engaged and committed to tackling this issue*.

The *Women for Gender Equality group* will continue to advocate for key reforms that reflect the priorities for *all women* at this time. It builds on the excellent work of *all those women currently committed* to achieving gender equality. While there is no ‘silver bullet’, we know that *working with women and girls* to promote gender equality contributes to achieving a host of health and developmental outcomes, not just those within the workplace.

I bring to my role as the Gender Equality Commissioner a sense of responsibility, obligation, and a deep commitment in my heart to *serve the women of this world*, and strive to serve the gender equality movement to the best of my abilities. The time to act is now.”


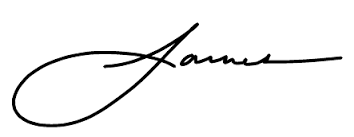


**Matthew Jamieson**

Gender Equality Commissioner

March 2016

**Manipulation Vignette 4: Government Agency Framing Gender Inequality as a Common Cause**

**Please read the below information carefully as we will be asking you a series of questions about it.**

**Social Inequality Study**

As you may be aware, gender inequality remains a key issue worldwide, particularly within the workplace. Women continue to earn less than men for equal work, and are less likely to be promoted to leadership positions compared to men. Below is some information about the Gender Equality Commission and a recent campaign it has held to combat gender inequality.

**About the Gender Equality Commission**

The Gender Equality Commission was appointed in March 2015. The Commission’s role is to address gender-based discrimination, sexual harassment, and other barriers to gender equality facing men and women across the world. As part of its role, in 2015 the Commission created the *Men and Women for Gender Equality* initiative which includes *50 of the world’s top male and female business and public sector leaders* striving to *achieve gender equality for men and women*. A milestone report has been released, detailing how the *Men and Women for Gender Equality group* are faring with their aspirations to increase the number of women in leadership positions within companies and decrease the gender pay gap.

**The following is an excerpt from the Gender Equality Commission’s recent press release:**

**Gender Equality Commission Calls for Immediate Action: “The time to act is now”**

“As you know, the *Men and Women for Gender Equality group* has just released its first annual report, *Gender Equality: What Matters to Men and Women*. The research for the report involved *extensive joint consultation with men and women* across the world. Our key finding is that *gender equality matters to both men and women* – but that progress towards *this common goal* has stalled. Our report shows gender inequality continues to be a *significant social and economic issue facing everyone* across the world, which is why it is *vital men and women are engaged and committed to tackling this issue together*.

The *Men and Women for Gender Equality group* will continue to advocate for key reforms that reflect the priorities for *all individuals* at this time. It builds on the excellent work of *all those men and women currently committed* to achieving gender equality. While there is no ‘silver bullet’, we know that *men and boys working together with women and girls* to promote gender equality contributes to achieving a host of health and developmental outcomes, not just those within the workplace.

We bring to our role as the Gender Equality Commission a sense of responsibility, obligation, and a deep commitment in our hearts to *serve the men and women of this world*, and strive to serve the gender equality movement to the best of our abilities. The time to act is now.”

Gender Equality Commission

March 2016

**Manipulation Vignette 5: Female Leader Framing Gender Inequality as a Common Cause**

**Please read the below information carefully as we will be asking you a series of questions about it.**

**Social Inequality Study**

As you may be aware, gender inequality remains a key issue worldwide, particularly within the workplace. Women continue to earn less than men for equal work, and are less likely to be promoted to leadership positions compared to men. Below is some information about the Gender Equality Commissioner and a recent campaign she has held to combat gender inequality.

**About the Gender Equality Commissioner**

Margaret Jamieson was appointed Gender Equality Commissioner in March 2015. The Commissioner’s role is to address gender-based discrimination, sexual harassment, and other barriers to gender equality *facing men and women* across the world. As part of her role, in 2015 the Commissioner created the *Men and Women for Gender Equality* initiative which includes *50 of the world’s top male and female business and public sector leaders* striving to achieve gender equality for men and women. A milestone report has been released, detailing how the Men and Women for Gender Equality group are faring with their aspirations to increase the number of women in leadership positions within companies and decrease the gender pay gap.

**The following is an excerpt from the Gender Equality Commissioner’s recent press release:**

**Gender Equality Commissioner Calls for Immediate Action: “The time to act is now”**

“As you know, the Men and Women for Gender Equality group has just released its first annual report, Gender Equality: What Matters to Men and Women. The research for the report involved *extensive joint consultation with men and women* across the world. Our key finding is that *gender equality matters to both men and women* – but that progress towards *this common goal* has stalled. Our report shows gender inequality continues to be a *significant social and economic issue facing everyone* across the world, which is why it is *vital men and women are engaged and committed to tackling this issue together*.

The *Men and Women for Gender Equality group* will continue to advocate for key reforms that reflect the priorities for *all individuals* at this time. It builds on the excellent work of *all those men and women currently committed* to achieving gender equality. While there is no ‘silver bullet’, we know that *men and boys working together with women and girls* to promote gender equality contributes to achieving a host of health and developmental outcomes, not just those within the workplace.

I bring to my role as the Gender Equality Commissioner a sense of responsibility, obligation, and a deep commitment in my heart to *serve the men and women of this world*, and strive to serve the gender equality movement to the best of my abilities. The time to act is now.”


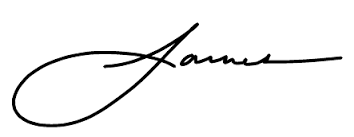


**Margaret Jamieson**

Gender Equality Commissioner

March 2016

**Manipulation Vignette 6: Male Leader Framing Gender Inequality as a Common Cause**

**Please read the below information carefully as we will be asking you a series of questions about it.**

**Social Inequality Study**

As you may be aware, gender inequality remains a key issue worldwide, particularly within the workplace. Women continue to earn less than men for equal work, and are less likely to be promoted to leadership positions compared to men. Below is some information about the Gender Equality Commissioner and a recent campaign he has held to combat gender inequality.

**About the Gender Equality Commissioner**

Matthew Jamieson was appointed Gender Equality Commissioner in March 2015. The Commissioner’s role is to address gender-based discrimination, sexual harassment, and other barriers to gender equality *facing men and women* across the world. As part of his role, in 2015 the Commissioner created the *Men and Women for Gender Equality* initiative which includes *50 of the world’s top male and female business and public sector leaders* striving to *achieve gender equality for men and women*. A milestone report has been released, detailing how the *Men and Women for Gender Equality group* are faring with their aspirations to increase the number of women in leadership positions within companies and decrease the gender pay gap.

**The following is an excerpt from the Gender Equality Commissioner’s recent press release:**

**Gender Equality Commissioner Calls for Immediate Action: “The time to act is now”**

“As you know, the *Men and Women for Gender Equality group* has just released its first annual report, *Gender Equality: What Matters to Men and Women*. The research for the report involved *extensive joint consultation with men and women* across the world. Our key finding is that *gender equality matters to both men and women* – but that progress towards *this common goal* has stalled. Our report shows gender inequality continues to be a *significant social and economic issue facing everyone* across the world, which is why it is *vital men and women are engaged and committed to tackling this issue together*.

The *Men and Women for Gender Equality group* will continue to advocate for key reforms that reflect the priorities for *all individuals* at this time. It builds on the excellent work of *all those men and women currently committed* to achieving gender equality. While there is no ‘silver bullet’, we know that *men and boys working together with women and girls* to promote gender equality contributes to achieving a host of health and developmental outcomes, not just those within the workplace.

I bring to my role as the Gender Equality Commissioner a sense of responsibility, obligation, and a deep commitment in my heart to *serve the men and women of this world*, and strive to serve the gender equality movement to the best of my abilities. The time to act is now.”


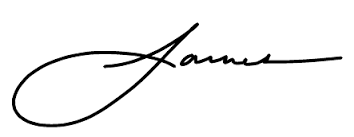


**Matthew Jamieson**

Gender Equality Commissioner

March 2016

**Study 1 List of Dependent Measures**

| **Even though you may not be familiar with the Commission, we would like you to tell us about your first impressions. Thinking of the gender equality movement and people who support it, would you say the Commission:**   |  | | --- | |  | | | | | | | |
| --- | --- | --- | --- | --- | --- | --- | --- | --- |
| | ***[Leader Prototypicality Scale - adapted from Platow & van Knippenberg’s (2001) Leader Relative Ingroup Prototypicality Scale]*** | | | | | | --- | --- | --- | --- | --- | |  | | *Strongly Neither Agree Strongly*  *Disagree nor Disagree Agree* | | | |  |  | |  |  | | Represents what is characteristic about members of the movement | | **1 2 3 4 5 6 7** | | | |  | |  | | | | Is representative of members of the movement | | **1 2 3 4 5 6 7** | | | |  | |  | | | | Is a good example of the kind of people who are involved in the movement | | **1 2 3 4 5 6 7** | | | |  | |  | | | | Stands for what people in the movement have in common | | **1 2 3 4 5 6 7** | | | |  | |  | | | | **[REVERSE SCORED]** Is not representative of the kind of people who are involved in the movement | | **1 2 3 4 5 6 7** | | | |  | |  | | | | Is very similar to most people in the movement | | **1 2 3 4 5 6 7** | | | | | | | | | |
|  | | | | | | |
|  | | |  | | | |
| ***[Leader Legitimacy Scale – supervisor/student’s own items]*** | | | | | | |
| **Based on the information you read, do you think the Gender Equality Commission’s statement was:** | | | | | | |
|  | | | *Strongly Neither Agree Strongly*  *Disagree nor Disagree Agree* | | | |
|  | |  | |  |  | |
| Legitimate | | | **1 2 3 4 5 6 7** | | | |
|  | | |  | | | |
| Justified | | | **1 2 3 4 5 6 7** | | | |
|  | | |  | | | |
| Valid | | | **1 2 3 4 5 6 7** | | | |
|  | | |  | | | |
| Reasonable | | | **1 2 3 4 5 6 7** | | | |
|  | | |  | | | |
|  | | |  | | | |
| ***[Leader Influence Scale – adapted from Wiley et al.’s (2013) Credibility Scale, and supervisor/student’s own items]*** | | | | | | |
| **Based on the information you read, do you think the Gender Equality Commission’s statement was:** | | | | | | |
|  | | | *Strongly Neither Agree Strongly*  *Disagree nor Disagree Agree* | | | |
|  | |  | |  |  | |
| Persuasive | | | **1 2 3 4 5 6 7** | | | |
|  | | |  | | | |
| Convincing | | | **1 2 3 4 5 6 7** | | | |
|  | | |  | | | |
| Compelling | | | **1 2 3 4 5 6 7** | | | |
|  | | |  | | | |
| Credible | | | **1 2 3 4 5 6 7** | | | |
|  | | |  | | | |
|  | ***[Collective Action Intentions Scale– adapted from Glasford & Calcagno’s (2012) Political Solidarity Subscale, combined with adapted version of van Zomeren, Spears, Fischer, & Leach’s (2004) Collective Action Tendencies Scale, and supervisor/student’s own items]*** | | | | |  |
| **Imagine you were approached by the Commission and asked to participate in their latest campaign for gender equality. In response, would you be willing to:** | | | | | | |
|  | | | *Strongly Neither Agree Strongly*  *Disagree nor Disagree Agree* | | | |
|  | |  | |  |  | |
| Sign a petition to stop inequality against women | | | **1 2 3 4 5 6 7** | | |  |
|  | | |  | | |  |
| Write a letter to the Prime Minister raising the issue of gender inequality | | | **1 2 3 4 5 6 7** | | |  |
|  | | |  | | |  |
| Talk to male colleagues about gender inequality | | | **1 2 3 4 5 6 7** | | |  |
|  | | |  | | |  |
| Talk to female colleagues about gender inequality | | | **1 2 3 4 5 6 7** | | |  |
|  | | |  | | |  |
| Participate in a demonstration against inequality on behalf of women | | | **1 2 3 4 5 6 7** | | |  |
|  | | |  | | |  |
| Participate in raising awareness about the injustices facing women | | | **1 2 3 4 5 6 7** | | |  |
|  | | |  | | |  |
| Do something together with other people to stop gender inequality | | | **1 2 3 4 5 6 7** | | |  |
|  | | |  | | |  |
| Participate in some form of collective action to stop gender inequality | | | **1 2 3 4 5 6 7** | | |  |

|  |  |
| --- | --- |
|  |  |
|  |  |
|  | |

| **In order to facilitate and improve our research, we want to ensure the information you read at the beginning of the survey was clear and easy to understand. In order to do so, without referring back to the information you read, please answer the following questions by circling your response:** | | | | | | | | | | | |  | | | |
| --- | --- | --- | --- | --- | --- | --- | --- | --- | --- | --- | --- | --- | --- | --- | --- |
|  | | | |  | | | | | | |  | | | | |
| The gender of the Commission/er was: | | | Male | | | | Female Not Stated | | | | |  | | | |
|  | | |  | | | | | | | | | | |  | |
|  | | | |  | | | | | | |  | | | | |
| **Thinking carefully about the information you read and without referring back to it, to what extent did you feel it provided information regarding (Note that 1 = Not at all and 7 = Very much so):** | | | | | | | | | | | |  | | | |
|  | | | *Not at Somewhat Very*  *all much so* | | | | | | | | | | |  | |
|  | |  | | | |  | | | |  | | | | |  |
| The need for *women alone* to stand up for equality | | | **1 2 3 4 5 6 7** | | | | | | | | | | |  | |
|  | | |  | | | | | | | | | | |  | |
| The need for *both men and women* to stand up for equality | | | **1 2 3 4 5 6 7** | | | | | | | | | | |  | |
|  | | |  | | | | | | | | | | |  | |
| Inequality being a *women’s only* issue | | | **1 2 3 4 5 6 7** | | | | | | | | | | |  | |
|  | | |  | | | | | | | | | | |  | |
| Inequality being a *men’s and women’s* issue | | | **1 2 3 4 5 6 7** | | | | | | | | | | |  | |
| ***[Demographic Information Items]*** | | | | | | | | | | | | |  | | |
| **Please specify the gender you identify as:** | Male | | | | Female | | | | Other (Please specify) __________________ | | | | | | |
|  | | | | | | | | | | | | | | | |
| **What is your age?** | _______________ | | | | | | | | | | | | | | |
|  |  | | | |  | | | |  | | | | | | |
| **What is your country of residence?** | Australia | | | | America Canada | | | | England | | | | | | |
|  | Other (Please specify) __________________ | | | |  | | | |  | | | | | | |
|  |  | | | |  | | | |  | | | | | | |
|  |  | | | | | | | | | | | | | | |
|  |  | | | | | | | | | | | | | | |
| **Please specify your current student status:** | Not currently studying | | | | Studying Full-time – Domestic Student | | | Studying Part-time – Domestic Student | | | | | | | |
|  |  | | | |  | | |  | | | | | | | |
|  | Studying Full-time – International Student | | | | Studying Part-time – International Student | | | Other (Please specify)  ___________________ | | | | | | | |
|  |  | | | | | | | | | | | | | | |
| **Please specify your current employment status:** | Employed Full-time | | | | Employed Part-time | | | Employed Casually | | | | | | | |
|  | Unemployed | | | | Other (Please specify)  ___________________ | | |  | | | | | | | |
|  |  | | | |  | | |  | | | | | | | |
|  | | |  | | | | | | | | | | |  | |
