# Supplementary material for: Whose Issue Is It Anyway? The Effects of Leader Gender and Equality Message Framing on Men’s and Women’s Mobilization Toward Workplace Gender Equality
Source: Front Psychol. 2018 Dec 11;9:2497. doi: 10.3389/fpsyg.2018.02497 (PMC6298257; doi:10.3389/fpsyg.2018.02497)
Supplement: Supplementary file 2 [file Table_2.DOC]

**Study 2 Manipulation Vignettes**

Leader Gender manipulations are underlined *Message Framing manipulations are italicised*

**Manipulation Vignette 1: Female Leader Framing Gender Inequality as a Common Cause**

**Please read the below information carefully as we will be asking you a series of questions about it.**

**Social Inequality Study**

As you may be aware, gender inequality remains a key issue worldwide, particularly within the workplace. Women are less likely to be promoted to leadership positions, and accumulate less retirement savings compared to men. Although significant progress has been made towards achieving equality over the past few decades, women still comprise only 21% of board members and 9% of CEOs globally.

**About the Chief Delegate to the Organization for Economic Co-Operation and Development**

Margaret Jamieson was appointed Chief Delegate to the OECD in March 2016. Part of her role is to address pay and leadership disparities within workplaces across the world. A milestone report has been released, detailing how the Chief Delegate is faring with her aspirations to increase the number of women in business leadership positions and decrease the retirement savings gap.

**The following is an excerpt from the Chief Delegate’s recent press release:**

**Chief Delegate to the Organization for Economic Co-Operation and Development Calls for Action**

*“As you know, I’ve just released my Department’s first annual report, the research for which involved extensive joint consultation with men and women across the globe. While gender inequality continues to be a significant social and economic issue, it is now an issue that matters to both men and women. However, our report shows that progress towards this common goal has stalled, which is why it’s important that both parties are engaged and committed to tackling this issue together. Admittedly, while there is no ‘silver bullet’, we know that men and boys working together with women and girls to promote gender equality contributes to achieving a host of health and developmental outcomes, not just those within the business world.”*


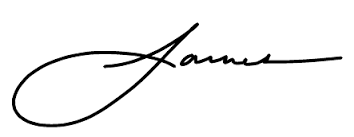


**Margaret Jamieson**

**Chief Delegate to the Organization for Economic Co-Operation and Development**

**April 2017**

**Manipulation Vignette 2: Female Leader Framing Gender Inequality as a Meritocracy Issue**

**Please read the below information carefully as we will be asking you a series of questions about it.**

**Social Inequality Study**

As you may be aware, gender inequality remains a key issue worldwide, particularly within the workplace. Women are less likely to be promoted to leadership positions, and accumulate less retirement savings compared to men. Although significant progress has been made towards achieving equality over the past few decades, women still comprise only 21% of board members and 9% of CEOs globally.

**About the Chief Delegate to the Organization for Economic Co-Operation and Development**

Margaret Jamieson was appointed Chief Delegate to the OECD in March 2016. Part of her role is to address pay and leadership disparities within workplaces across the world. A milestone report has been released, detailing how the Chief Delegate is faring with her aspirations to increase the number of women in business leadership positions and decrease the retirement savings gap.

**The** **following is an excerpt from the Chief Delegate’s recent press release:**

**Chief Delegate to the Organization for Economic Co-Operation and Development Calls for Action**

*As you know, I’ve just released my Department’s first annual report. While gender inequality continues to be a significant social and economic issue, those women who* are *in senior management roles show that it is possible to move up the leadership ladder by working hard, ‘leaning in’, and making sacrifices. These women demonstrate that all individuals can succeed in the workplace irrespective of their gender — as long as they are prepared to invest the time, energy, and significant effort needed for such advancement. Indeed, in the business world, those who apply themselves and make sacrifices along the way reap the rewards, because business — and society more broadly — has always rewarded hard work.”*


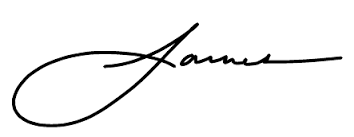


**Margaret Jamieson**

**Chief Delegate to the Organization for Economic Co-Operation and Development**

**April 2017**

**Manipulation Vignette 3: Male Leader Framing Gender Inequality as a Common Cause**

**Please read the below information carefully as we will be asking you a series of questions about it.**

**Social Inequality Study**

As you may be aware, gender inequality remains a key issue worldwide, particularly within the workplace. Women are less likely to be promoted to leadership positions, and accumulate less retirement savings compared to men. Although significant progress has been made towards achieving equality over the past few decades, women still comprise only 21% of board members and 9% of CEOs globally.

**About the Chief Delegate to the Organization for Economic Co-Operation and Development**

Matthew Jamieson was appointed Chief Delegate to the OECD in March 2016. Part of his role is to address pay and leadership disparities within workplaces across the world. A milestone report has been released, detailing how the Chief Delegate is faring with his aspirations to increase the number of women in business leadership positions and decrease the retirement savings gap.

**The following is an excerpt from the Chief Delegate’s recent press release:**

**Chief Delegate to the Organization for Economic Co-Operation and Development Calls for Action**

*“As you know, I’ve just released my Department’s first annual report, the research for which involved extensive joint consultation with men and women across the globe. While gender inequality continues to be a significant social and economic issue, it is now an issue that matters to both men and women. However, our report shows that progress towards this common goal has stalled, which is why it’s important that both parties are engaged and committed to tackling this issue together. Admittedly, while there is no ‘silver bullet’, we know that men and boys working together with women and girls to promote gender equality contributes to achieving a host of health and developmental outcomes, not just those within the business world.”*


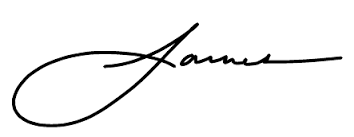


**Matthew Jamieson**

**Chief Delegate to the Organization for Economic Co-Operation and Development**

**April 2017**

**Manipulation Vignette 4: Male Leader Framing Gender Inequality as a Meritocracy Issue**

**Please read the below information carefully as we will be asking you a series of questions about it.**

**Social Inequality Study**

As you may be aware, gender inequality remains a key issue worldwide, particularly within the workplace. Women are less likely to be promoted to leadership positions, and accumulate less retirement savings compared to men. Although significant progress has been made towards achieving equality over the past few decades, women still comprise only 21% of board members and 9% of CEOs globally.

**About the Chief Delegate to the Organization for Economic Co-Operation and Development**

Matthew Jamieson was appointed Chief Delegate to the OECD in March 2016. Part of his role is to address pay and leadership disparities within workplaces across the world. A milestone report has been released, detailing how the Chief Delegate is faring with his aspirations to increase the number of women in business leadership positions and decrease the retirement savings gap.

**The following is an excerpt from the Chief Delegate’s recent press release:**

**Chief Delegate to the Organization for Economic Co-Operation and Development Calls for Action**

*As you know, I’ve just released my Department’s first annual report. While gender inequality continues to be a significant social and economic issue, those women who* are *in senior management roles show that it is possible to move up the leadership ladder by working hard, ‘leaning in’, and making sacrifices. These women demonstrate that all individuals can succeed in the workplace irrespective of their gender — as long as they are prepared to invest the time, energy, and significant effort needed for such advancement. Indeed, in the business world, those who apply themselves and make sacrifices along the way reap the rewards, because business — and society more broadly — has always rewarded hard work.”*


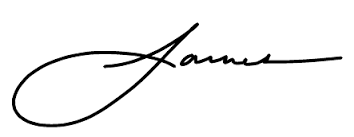


**Matthew Jamieson**

**Chief Delegate to the Organization for Economic Co-Operation and Development**

**April 2017**

**Study 2 List of Dependent Measures**

|  | **Even though you may not be familiar with the Chief Delegate and their initiative, we are interested in your first impressions. Thinking of the gender equality movement and people who support it, would you say the Chief Delegate:** | | | | | | | | | | | | | |
| --- | --- | --- | --- | --- | --- | --- | --- | --- | --- | --- | --- | --- | --- | --- |
|  | ***[Leader Prototypicality Scale - adapted from Platow & van Knippenberg’s (2001) Leader Relative Ingroup Prototypicality Scale]*** | | | | | | | | | | | | | |
|  |  | | |  | | | |  | | |  | | | |
|  |  | *Strongly Neither Agree Strongly*  *Disagree nor Disagree Agree* | | | | | | | | | | | | |
|  | Represents what is characteristic about members of the movement | **1 2 3 4 5 6 7** | | | | | | | | | | | | |
|  | Represents members of the movement | **1 2 3 4 5 6 7** | | | | | | | | | | | | |
|  | Is a good example of the kind of people who are involved in the movement | **1 2 3 4 5 6 7** | | | | | | | | | | | | |
|  | Stands up for what people in the movement have in common | **1 2 3 4 5 6 7** | | | | | | | | | | | | |
|  | Is very similar to most people in the movement | **1 2 3 4 5 6 7** | | | | | | | | | | | | |
|  | **Thinking about the information you read, to what extent do you think that the Chief Delegate and their statement is:** | | | | | | | | | | | |  | |
|  | ***[Leader Legitimacy Scale – supervisor/student’s own items]*** | | | | | | | | | | | |  | |
|  |  | | | | |  | | | |  | |  |  | |
|  | **Legitimate** | | **1 2 3 4 5 6 7** | | | | | | | | | |  | |
|  | **Justified** | | **1 2 3 4 5 6 7** | | | | | | | | | |  | |
|  | **Valid** | | **1 2 3 4 5 6 7** | | | | | | | | | |  | |
|  | **Reasonable** | | **1 2 3 4 5 6 7** | | | | | | | | | |  | |
|  | ***[Leader Influence Scale – adapted from Wiley et al.’s (2013) Credibility Scale, and supervisor/student’s own items]*** | | | | | | | | | | | |  | |
|  |  | | | | |  | | | |  | |  |  | |
|  | **Persuasive** | | **1 2 3 4 5 6 7** | | | | | | | | | |  | |
|  | **Convincing** | | **1 2 3 4 5 6 7** | | | | | | | | | |  | |
|  | **Compelling** | | **1 2 3 4 5 6 7** | | | | | | | | | |  | |
|  | **Credible** | | **1 2 3 4 5 6 7** | | | | | | | | | |  | |
| **Imagine that the Chief Delegate has approached you directly to help with their campaign for gender equality. In that context, please rate the extent to which you agree with the following statements:** | | | | | | | | | | | | | |  |
| ***[Collective Action Intentions Scale– adapted from Calogero’s (2013) Collective Action Scale, and Subasic, Hardacre, Elton, Branscombe, Ryan, & Reynolds’ (2018)*** | | | | | | | | | | | | | |  |
|  | | | | | |  | | | |  | |  | |  |
|  | | | | | *Strongly Neither Agree Strongly*  *Disagree nor Disagree Agree* | | | | | | | | |  |
| I would discuss issues related to gender equality with friends or colleagues in person or online (e.g., email, Facebook, Twitter, etc | | | | | **1 2 3 4 5 6 7** | | | | | | | | |  |
| I would participate in a demonstration against systematic gender inequality | | | | | **1 2 3 4 5 6 7** | | | | | | | | |  |
| I would sign a petition (in person or online) in support of women’s rights and gender equality | | | | | **1 2 3 4 5 6 7** | | | | | | | | |  |
| I would vote for a political party that fights against gender inequality | | | | | **1 2 3 4 5 6 7** | | | | | | | | |  |
| I would contact my local member of parliament/congress to urge them to support legislation addressing gender disparities | | | | | **1 2 3 4 5 6 7** | | | | | | | | |  |
|  | | | | |  | | | | | | | | |  |
| I would tweet or post on social media about gender inequality | | | | | **1 2 3 4 5 6 7** | | | | | | | | |  |
| To show that you are paying attention, please select ‘Strongly AGREE’ (i.e., 7) | | | | | **1 2 3 4 5 6 7** | | | | | | | | |  |
| **Please rate the extent to which you agree with the following statements:** | | | | | | | | | | | | | |  |
| ***[Common Cause Scale – adapted from Subasic, Hardacre, Elton, Branscombe, Ryan, & Reynolds’ (2018) Common Cause Scale]*** | | | | | | | | | | | | | |  |
| I feel solidarity with the women affected by income inequality and leadership disparities | | | | | **1 2 3 4 5 6 7** | | | | | | | | |  |
| The women calling for action on this issue reflect the values that I consider to be important | | | | | **1 2 3 4 5 6 7** | | | | | | | | |  |
| I see myself as someone who shares the views of the women who object to these forms of inequality | | | | | **1 2 3 4 5 6 7** | | | | | | | | |  |
|  | | | | | | | | |  | | | | |  |
| Those seeking to reduce income inequality and leadership disparities between men and women share my goals and concerns | | | | | | | **1 2 3 4 5 6 7** | | | | | | |  |
